# Assessment of Drinking Water Quality in Urban Water Supply Systems: The Case of Hawassa City, Ethiopia

**DOI:** 10.1155/2023/8880601

**Published:** 2023-08-14

**Authors:** Yirged Antehun Mengstie, Wendesen Mekonin Desta, Esayas Alemayehu

**Affiliations:** ^1^Institute of Technology Faculty of Biosystem and Water Resource Engineering, Department of Water Supply and Environmental Engineering, Hawassa University, Hawassa, Ethiopia; ^2^Institute of Water and Energy Science (Including Climate Change), Pan African University, Tlemcen, Algeria; ^3^Jimma Institute of Technology, Faculty of Civil and Environmental Engineering, Department of Water Supply and Environmental Engineering, Jimma University, Jimma, Ethiopia; ^4^Africa Center of Excellence for Water Management, Addis Ababa University, P.O. Box-1176, Addis Ababa, Ethiopia

## Abstract

In many developing countries, such as Ethiopia, water quality and the risk of water-related diseases are serious public health issues. The present study goal was to assess the drinking water quality from source to household tap water. To characterize and analyze drinking water quality parameters, 21 water samples were collected, of which 11 water samples were collected from sources (spring, borehole, and river), 4 from service reservoirs, and 6 from tap water. The mean values of the parameters were as follows: total dissolved solids (TDS) (142.79 mg/L), temperature (22.08°C), turbidity (9.49 NTU), electrical conductivity (EC) (250.14°*μ*S/cm), pH (7.45 mg/L), fluoride (1.15 mg/L), nitrate (NO_3_^−^) (2.91 mg/L), total hardness (TH) (57.45 mg/L), calcium (41.7 6 mg/l), magnesium (10.74 mg/L), phosphate (0.44 mg/L), sulfate (3.99 mg/L), residual chlorine (1.53 mg/L), alkalinity (196.39 mg/L), and microbiological (total coliform and coliform/CFU) which were the main physiochemical parameters analyzed for the study. The findings revealed that the majority of the water quality parameters tested were within the WHO and National Drinking Water Quality Standards (NDWQS). However, some of the parameters such as temperature, turbidity, fluoride, and residual chlorine did not meet the standards. The mean temperatures at the source, reservoir, and tap water were 22.01°C 22.5°C,and 21.83°C, respectively. Turbidity levels in source samples ranged from 10 to 45 NTU, with a mean of 24.5 NTU, exceeding the WHO's recommendation of less than 5 NTU. The Boko Alamura well had a high fluoride content (3.9 mg/l), which was above the WHO and NDWQS permissible limits. There was no free residual chlorine in the tap water sample. The results show that the Hawassa drinking water supply did not contain total or fecal coliform in any of the samples tested. The overall WQI for the water source, reservoir, and tap water was also determined to be 89, 71, and 69.7 points, respectively. Therefore, based on the WQI result, Hawassa drinking water quality is good for the source, reservoir, and tap water.

## 1. Introduction

Water is a natural resource that is critical to human survival[1–3]. It sustains all forms of life and generates jobs and wealth in the water, tourism, and recreation industries. The global slogan “Water is Life” implies that water is one of the most basic human needs. Life as we know it on our planet would be impossible without water [4, 5]. Water distribution networks are critical in modern communities because their proper operation is directly related to the well-being of the population [6, 7]. In spite of such importance, water crises and quality are major concerns in many countries, particularly in arid and semiarid regions where water scarcity is common, and water quality assessment has received little attention [8–10]. Water quality is constantly under attack because it is essential to the human body and ecosystem. Globally, the growing human population has a negative impact on surface waters and watersheds. As the demand for freshwater rises with the growth of the human population, the degradation of the water quality in aquatic ecosystems has become a global concern [11]. Although urbanization is a global phenomenon with far-reaching consequences for natural ecosystems, the ecosystem's primary constituent is water, a valuable natural resource and national asset. Water sources include rivers, lakes, glaciers, rainwater, groundwater, and so on. Water resources are important in many sectors of the economy, including agriculture, livestock production, forestry, industrial activities, hydropower generation, fisheries, and other creative activities [12]. In order to achieve the desired goal, it is crucial to use a variety of physical, chemical, and biological variables for different purposes (drinking, industrial, agricultural, recreational, and habitat). [13, 14]. Groundwater is a critical component of human development because it is the primary source of drinking water in many countries around the world [15–17]. The insufficiency in surface water resources makes the people dependent on groundwater for the regular water supply [18]. Monitoring water quality is an essential tool in the management of freshwater resources. The International Organization for Standardization (ISO) defines monitoring as “the programmed process of sampling, measurement, and subsequent recording or signaling, or both, of various water characteristics, frequently with the goal of assessing conformity to specified objectives.” The most popular definition of water quality is “it is the physical, chemical, and biological characteristics of water” [19]. It was prudent to conduct research on the city's water supply system in order to determine the quality of drinking water.

Several major issues affecting human survival on Earth are caused by a lack of clean water for a large number of communities, as well as environmental aesthetics [20, 21]. In many developing countries, such as Ethiopia, water quality and the risk of water-related diseases are serious public health issues. It can be directly or indirectly linked to public health due to the low or high concentrations of numerous contaminants in drinking water [22–24]. Access to improved water supply and sanitation has been very low, and hence, the majority of communicable diseases are associated with unsafe and inadequate water supply [25]. In Ethiopia, the safety of potable water and the risk of waterborne diseases are major public health concerns. A communicable disease associated with unsafe and inadequate water and poor human excreta disposal accounts for approximately 60% of the health problem [26]. Waterborne diseases, particularly diarrhea, coliforms, and *E. coli* microorganisms, were prevalent in SNNP. This is because there was insufficient investigation and subsequent control of water quality parameters. Water-related diseases are frequently reported as being among the top ten diseases in the region's health sector, and there are several signs that the region's population is suffering from water-related diseases, most likely as a result of poor drinking water quality [27, 28]. Contamination can significantly change the chemical properties of water, compromising the overall balance of the system, causing economic losses, and making its consumption impractical [29–31].

So far, no research activity has been conducted on the city's drinking water quality that may enable us to know the quality of drinking water; however, it has been observed that some people in the study area did not drink tap water and complained that the water has a salty taste. They generally distrust the quality of tap water and prefer to drink bottled water. The main objectives of this study are to investigate the drinking water quality in Hawassa City utilizing on-site and laboratory experiments and to assess the findings by contrasting and comparing them to prior studies, national and international standards, and guidelines. Based on the aforementioned study goals, it not only assesses the safety of the source, reservoir, and tap water for consumption but also offers a foundation for their management strategy toward them.

## 2. Research Methodology

### 2.1. Description of the Study Area

**Figure 1:**
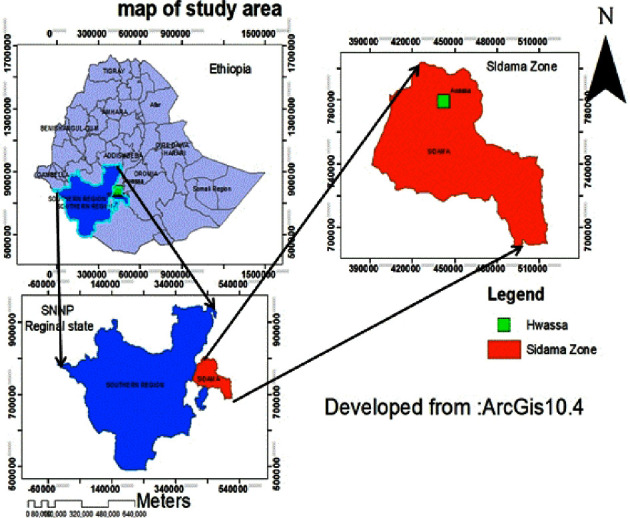
Map of the study area.

This study was carried out in Hawassa, a city in Ethiopia's Sidama regional state. The city is situated between 7°3′1.3464″N latitude and 38°29′43.8144″E longitude, at a height of 1708 meters above sea level. Addis Ababa is located 273 kilometers to the south of the city. The city is the capital of the Southern Nations, Nationalities, and Peoples' Region, as well as a special zone.  Figure 1 depicts the location of the study area.

### 2.2. Climate and Hydrology

Hawassa town has a hot temperature, fluctuating between 10°C in winter and 30°C in summer. The town average annual rainfall is 956 mm. The average maximum rainfall during the rainy season is about 126 mm in September. The number of sunny hours in a day ranges from 4 hours in the rainy season to 9 hours in the dry season. Relative humidity varies from 40% to 90% during the year. The average wind speed recorded ranges from 0.6 m/s to 1.1 m/s. According to the National Weather Service, the estimated annual PET intake for the Hawassa station is about 1599 mm, with a minimum of 102 mm in July and a maximum of 173 mm in December.

### 2.3. Geology and Hydrogeology

The Hawassa Basin is a volcanic tectonic collapse located in the central part of Ethiopia's main Rift Valley. There are several rift system faults that tend to the north and northeast along Lake Hawassa. These errors are extensive and often constitute step errors. They mainly dominate the south and southwest of the lake. The collapsing structure of the volcano forms an almost circular pattern around the Hawassa Lake Basin. This collapse intersects several Main Ethiopian Rift (MER) fault systems, suggesting that the collapse occurs after the fault.

Lake Hawassa covers an area of 100 square kilometers, while Cheleleka Wetland covers an area of 12 square kilometers. Recent lake and alluvial deposits, coal cones, rhyolite lava flows, and related igneous rocks, tuffs, and volcanic ash form this basin. Rhyolitic lava flows and related igneous and ash rocks belong to recent rhyolite volcanic centers and coal cones to basalt of recent highlands. The cliffs and mountains at the eastern edge of the Hawassa Lake Basin comprise the Nazareth Series, consisting of ignimbrite, unwanted tuff, ash stream, rhyolitic stream, dome, and trachyte. The northern, southwestern, and western margins include the Dino Formation, which is characterized by lava rock overlaid by coarse pumice of tuff ignimbrite with a rare alternation of lake sediments. The Hawassa Basin strata are based on the Dino Formation, also known as the Nazareth Series.

### 2.4. Data Collection Process

Personal observation and field measurement were used to collect data. This was accomplished by employing the primary data collection method to obtain the information required to meet the objective. On both primary and secondary data, qualitative and quantitative analyses were performed. Tables, maps, and/or phrases were used to evaluate the data qualitatively. In contrast, quantitative data were analyzed in Excel.

### 2.5. Sampling Methods

Samples were collected from raw water source locations such as reservoirs and water taps where customers receive water. The tap water sample was collected twice, from two different kebeles (it is collected randomly from ketena one and two of the kebele). The total sample was collected in three phases. In the first phase (10/12/2013 E. C to 18/12/2013 E. C), 11 water samples were collected from sources. In the second phase (18-19/12/2013 E. C), the samples were collected from the reservoir.

**Figure 2:**
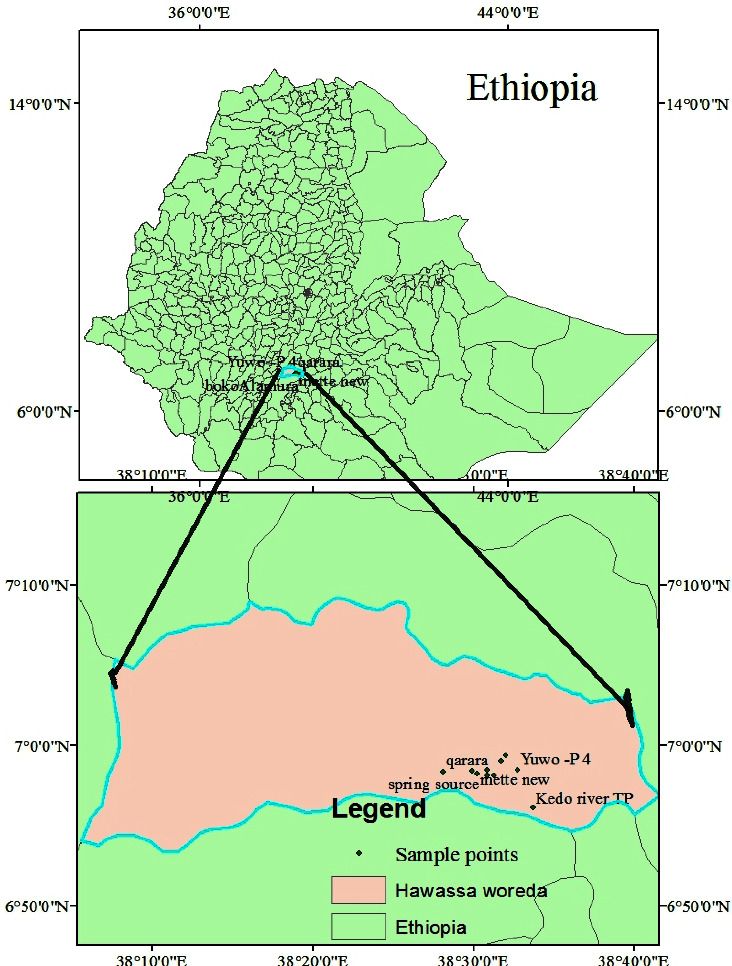
Sample coordinates for source.

In the third phase, the samples were collected from the water tap in 29/12/2013 E. C. Precautions were taken for sampling. Contaminant-free containers were used, devices or instruments used for sampling were calibrated, and the time and the type of samples were leveled. The location of the sampling points is shown in Figure 2.

### 2.6. Water Quality Parameter Analysis and Instruments

Water samples were collected from Hawassa's drinking water supply system's 21 drinking water supply stations. Four water samples were taken from service reservoirs, and three kebeles (small administrative) of water taps were also used to obtain six samples. Taps were turned on or left running for at least a few minutes prior to sampling to ensure a representative sample (temperature and electrical conductivity were monitored to verify this). The other 11 samples were collected from the source water. Various physicochemical parameters (electrical conductivity, TDS, pH, and temperature) of the water samples were measured in the field using portable meters at the time of sampling. Water samples were taken in clean containers provided by the laboratory.

### 2.7. Physicochemical Test Procedures

Sensitive water quality parameters such as temperature, pH, EC, and TDS were determined using on-site measurements. A thermometer and a portable digital pH meter were used to measure temperature and pH. The pH meter was calibrated with pH 4.0 and pH 7.0 before being used for the analysis, and it was washed with distilled water between samples as directed in the pH meter operation guide. A portable digital conductivity meter was used to measure electrical conductivity and total dissolved solids (TDS). Their measurements were taken immediately after the samples were collected at each location. The remaining indicators of water quality were measured in accordance with the standards. The equipment was thoroughly cleaned and disinfected before each use to prevent secondary contamination and ensure accurate results.

### 2.8. Bacteriological Parameter Analysis

To avoid the growth or death of microorganisms in the sample, bacteriological tests were done on the same day the sample was collected. Using the membrane filtration method, a 100 ml water sample was sucked through a filter with a little hand pump. After filtration, the bacteria on the filter paper were placed in a Petri dish with a nutritive solution (also known as culture media, broth, or agar). The temperature and period of incubation differed based on the type of indicator bacteria and culture media applied (for example, total coliforms were incubated at 35°C and fecal coliforms were cultured at 44.5°C with some types of culture media).

### 2.9. Calculation of Water Quality Index (WQI)

The water quality index (WQI) is a straightforward and effective method for determining water quality. It is also an excellent way to disseminate information about water quality. The WQI method is a straightforward and practical way to assess the general quality of surface/groundwater and its appropriateness as drinking water [32, 33]. The water quality index (WQI) is a measure of the acceptability of water for human consumption that takes into account the combined effects of various water quality factors [34]. It was calculated using the weighted arithmetic index method adopted from [35]. The quality rating scale for each parameter qi was calculated by using the following equation:

(1) $$\begin{array}{c} q_{i}=\left(\frac{C_{i}}{S_{i}}\right)\times 100\text{.} \end{array}$$

A quality rating scale (*q*_*i*_) for each parameter is assigned by dividing its concentration (*C*_*i*_) in each water sample by its respective standard (*S*_*i*_), and the result is multiplied by 100. The inversely proportional value of the recommended standard (*S*_*i*_) of the corresponding parameter is used to calculate the relative weight:

(2) $$\begin{array}{c} Wi=\frac{1}{Si}\text{.} \end{array}$$

The overall water quality index (WQI) was calculated by aggregating the quality rating (Qi) with unit weight (Wi) linearly:

(3) $$\begin{array}{c} WQI=\left(\overset{i-n}{\underset{i=1}{\sum }}Wiqi\right)\text{.} \end{array}$$

Generally, WQI is discussed for a specific and intended use of water. In this study, the WQI for drinking purposes is considered, and permissible WQI for the drinking water is taken as 100:

(4) $$\begin{array}{c} overall\;WQI=\left(\frac{\sum q_{i}w_{i}}{\sum w_{i}}\right)\text{.} \end{array}$$

## 3. Results and Discussion

### 3.1. Physicochemical Analysis Results of Source, Reservoir, and Tap Water Samples

The physicochemical parameters such as total dissolved solids (TDS), turbidity, electrical conductivity (EC), temperature, pH, calcium, magnesium, total hardness, alkalinity, fluoride, nitrate (NO_3_), sulfate (SO_4_), phosphate (PO_4_), and residual chlorine at different sample locations are shown in Table 1. Figures 3–15 depict the detailed analysis.

#### 3.1.1. Total Dissolved Solids (TDS)

**Figure 3:**
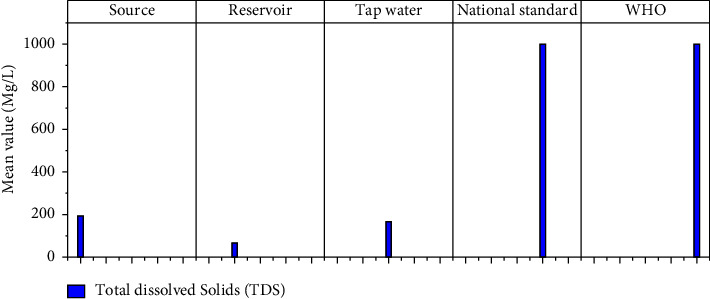
Total dissolved solid result of source, reservoir, and tap waters.

TDS in drinking water has no health-based limit. As a result, TDS occurs in drinking water at concentrations far below those that are harmful. Water with TDS levels less than 100 mg/L, on the other hand, is considered to be good in terms of palatability [36].  Figure 3 shows that the mean concentration of TDS in water samples in the study area ranged from 67.3 to 190.9 mg/l. The source has the highest TDS value (190.9 mg/l). TDS levels are higher in the source and water tap samples than in the reservoir samples. However, the health risks are minimal because the TDS value is much lower than 1,000 mg/l, which is the WHO and NDWQS maximum permissible limit. The TDS values of water in this study are higher than those in previous studies' results, i.e., the mean TDS records of various cities' water sources; the TDS at Nekemte is 48 mg/l, at Damot Sore Woreda is 67.79 mg/l, and at Tula subcity is 150.7 mg/l.

#### 3.1.2. Turbidity

**Figure 4:**
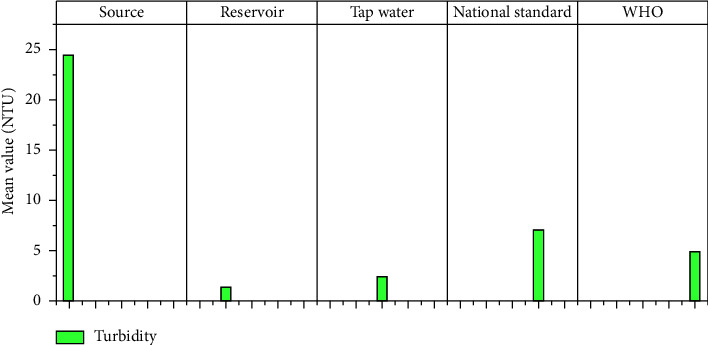
Mean turbidity of source, reservoir, and tap waters.

The turbidity levels in the source samples ranged from 10 to 45 NTU, with a mean of 24.5 NTU, which was higher than the WHO and NDWQS recommendation of 5 NTU and 7 NTU. The mean turbidity values at the reservoir and tap water, on the other hand, are determined to be within the permissible limits of 1.55 NTU and 2.48 NTU, respectively (Figure 4). Turbidity in water is caused by sewage matter, which increases the risk of pathogenic organisms being shielded by turbidity particles and thus escaping the disinfectant's effect.

#### 3.1.3. Electrical Conductivity (EC)

**Figure 5:**
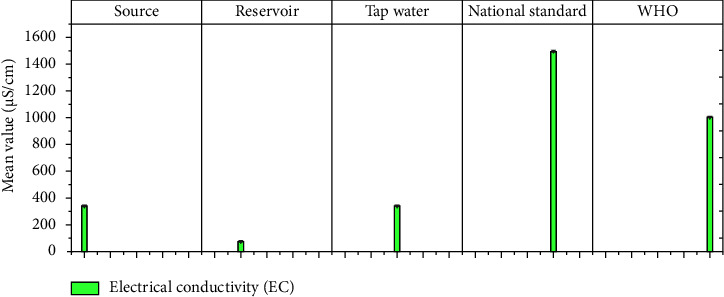
Mean electric conductivity values of source, reservoir, and tap waters.

Electrical conductivity (EC), a measure of water's ability to conduct an electric current, is proportional to the amount of dissolved minerals in the water but does not indicate which element is present. In contrast, a higher EC value indicates the presence of pollutants such as sodium, potassium, or chloride [37]. As shown in Figure 5, the samples from the Hawassa water source have a mean EC value of 339, with maximum and minimum values of 243 and 569 (*μ*S/cm). The Hawassa water reservoir's average EC is 72.75 *μ*S/cm, with a range of 35 to 115 *μ*S/cm. Similarly, Hawassa tap water has an average EC value of 338.67 *μ*S/cm, with a range of 166 to 388 *μ*S/cm. The tested values for Hawassa drinking water at the source and tap water are within permissible limits when compared to WHO and NDWQS standards.

#### 3.1.4. Temperature

**Figure 6:**
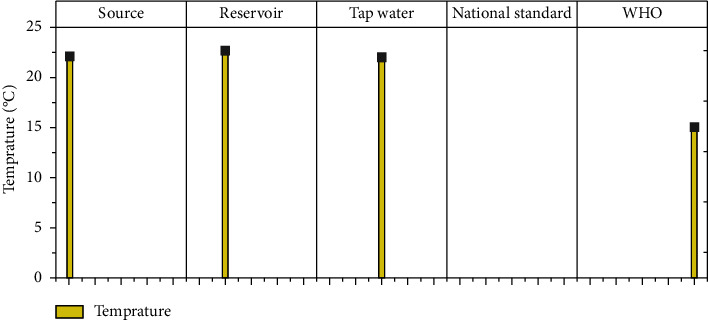
Mean temperature variation of source, reservoir, and tap waters.

Temperature is one of the physicochemical factors used to determine drinking water quality. As the temperature of the water rises, so does the demand for disinfectants and microbial activity, reducing the palatability of the water [25]. However, the results show that all of the temperature values for the Hawassa water samples from several samples are above the WHO recommended limit. The temperature range of the source was 21–22.8°C, which corresponded to the minimum and maximum temperatures of the water source. Similarly, the reservoir and tap water samples have temperatures ranging from 21 to 24°C and 21 to 23°C, respectively, which are outside of the acceptable temperature range set by the World Health Organization [36]. The majority of the sampled sites had temperature variations from the sources to the water taps, which did not meet the WHO requirement of 15°C. The reservoir (new reservoir 1) sample had the highest temperature (24°C) (Figure 6). The tropics have a hot climate with lots of rain, which may have contributed to the high temperatures found in water samples from various Ethiopian cities [38]. Similarly, earlier research in the Damot Sore Woreda of the south regional state [39] reported a mean temperature of 23.27°C.

#### 3.1.5. pH

**Figure 7:**
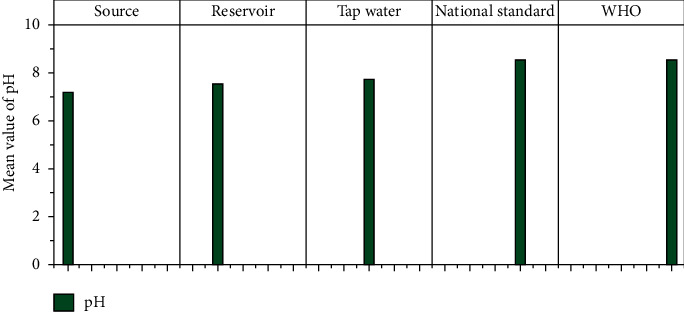
Mean pH of source, reservoir, and tap waters.

As a starting point for the pH scale, neutral chemicals are used. Alkaline or basic compounds have a pH greater than 7.0 (7.1–14.0). Acidic compounds have a pH value less than 7.0 (0–6.9). pH adjustment is a common method in water treatment and one of the most critical operational elements for water treatment processes such as disinfection and flocculation [40]. The WHO defines the minimum and maximum permissible pH for drinkable water as 6.5 to 8.5 [36]. All water samples had a pH range of 6.5–7.99, but the mean pH increased from source to tap water (Figure 7). There were no statistically significant differences between sampling stations, and the pH levels in this study area are within WHO and national guidelines.

#### 3.1.6. Calcium and Magnesium

**Figure 8:**
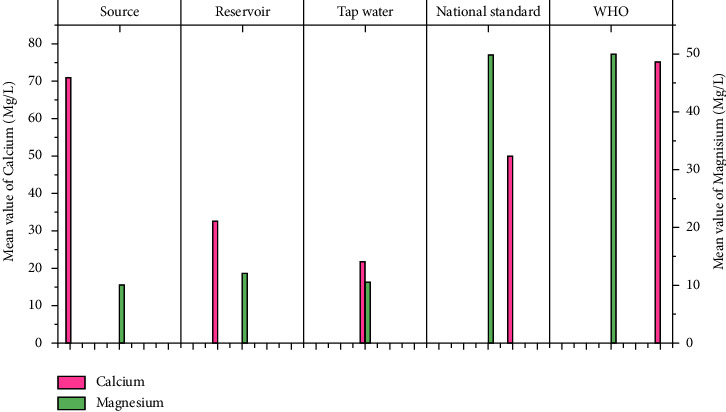
Mean Ca and Mg value for sampled water.

Calcium comes from both natural and man-made sources. Water that flows within an aquifer could be internal. The average calcium levels in the study's source, reservoir, and tap waters are 72.31 mg/l, 32.1 mg/l, and 21.3 mg/l, respectively (Figure 8). The maximum calcium value of the source water (Abella Wondo No. 2 well, 160 mg/l) does not meet the WHO's calcium limit for drinking water [36]. These variations could be caused by the geological contents of the well. All reservoir and tap water samples, on the other hand, are within the recommended level of 75 mg/l. Magnesium levels in this study's source, reservoir, and tap water samples were found to be 9.9 mg/l, 12 mg/l, and 10.33 mg/l, respectively (Figure 8). This means that the magnesium level is within an acceptable range and has no negative health implications.

#### 3.1.7. Total Hardness

**Figure 9:**
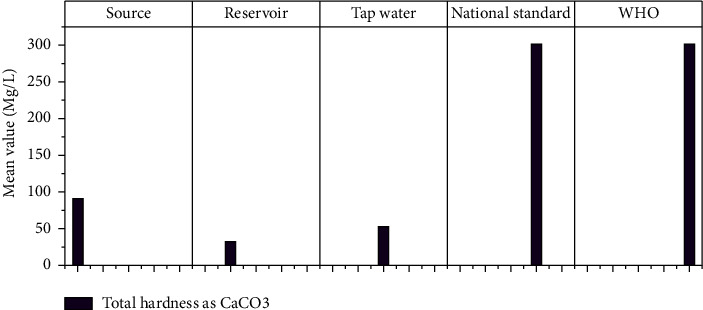
Total hardness mean values at different sampling sites.

It denotes the total amount of calcium and magnesium ions present in the body. Initially, hardness was measured and analyzed in raw water samples as a proxy for water quality in terms of precipitating soap. The highest permissible limit of total hardness as CaCO_3_, according to the World Health Organization [36], is 300 mg/l. The mean total hardness at the source, reservoir, and tap water is 89.86 mg/l, 30 mg/l, and 52.50 mg/l, respectively, according to the laboratory results of this study (Figure 9). According to WHO standards, the degree of hardness of the Hawassa City water supply is moderately soft, which is not harmful to users.

#### 3.1.8. Alkalinity

**Figure 10:**
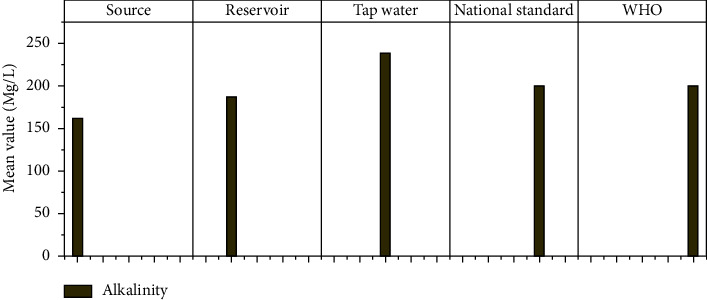
Alkalinity mean values at different sampling sites.

Water sources tolerate extremes in these ranges, with alkalinity values ranging from 5 to 125 mg/l considered normal. According to the WHO standard guideline for drinking water potability, the maximum acceptable permitted value of CaCO_3_ should not exceed 200 mg/l. According to laboratory test results, the total alkalinity of the Hawassa City water supply samples ranged from 124 to 280 mg/l of CaCO_3_ at the source sample, 125 mg/l to 230 mg/l at the reservoir sample, and 195 mg/l to 310 mg/l at the tap water sample (Figure 10). According to the findings of this study, one source sample, samples from new reservoir 1 and 2, and a sample from pissa kebele sample 2 did not meet the standards established.

#### 3.1.9. Fluoride

**Figure 11:**
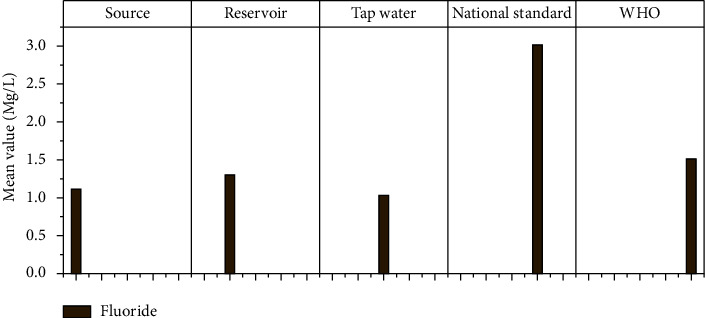
Fluoride mean values at different sampling sites.

The fluoride concentration in Hawassa City's water sources ranged from 0 to 3.9 mg/l (Figure 11). The fluoride concentration in the Boko Alamura well was 3.9 mg/l, which was higher than WHO and national standards. The WHO recommends a fluoride concentration of 1.5 mg/l, but Ethiopian drinking water recommendations require less than 3 mg/l [41]. Other water tests (reservoir and water tap samples) came up short of the acceptable limit. The fluoride levels in this study exceeded the maximum values of Damot Sore Woreda (1.13 mg/l) [5].

#### 3.1.10. Nitrate (NO_3_)

**Figure 12:**
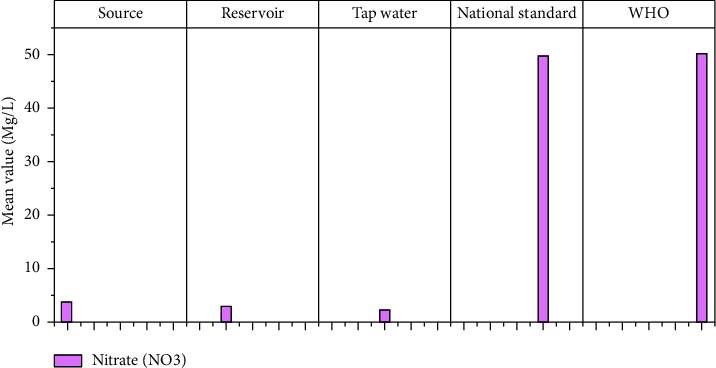
NO_3_ mean values at different sampling sites.

The main sources of nitrates in drinking water are fertilizer runoff, sewage leakage, and erosion of natural deposits [42, 43]. According to laboratory results, the mean nitrate levels of Hawassa's water source, reservoir, and water tap are 3.78, 2.73, and 2.23 mg/l, respectively (Figure 12). The WHO and Ethiopian standards were found to be met by all of the samples tested. Water with nitrate concentrations greater than 10 mg/l nitrate-N will cause methaemoglobinaemia in users, according to the guidelines [41]. As a result, referring to the guideline, there is no nitrate problem in Hawassa's drinking water supply, according to the findings.

#### 3.1.11. Sulfate (SO_4_)

**Figure 13:**
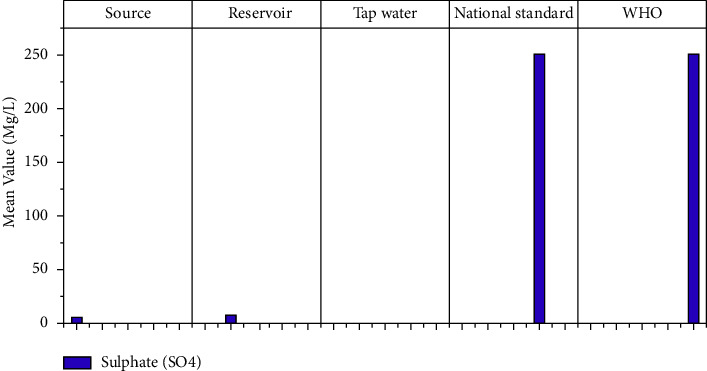
Sulfate mean values at different sampling sites.

Sulfates have no health-based recommendations. However, because drinking water with a high sulfate concentration can cause gastrointestinal effects, drinking water sources with a sulfate concentration of more than 500 mg/l should be reported to health authorities. Sulfate in drinking water can also cause a noticeable taste and contribute to distribution system corrosion [36]. The study's laboratory results show that the mean sulfate level in the Hawassa water supply's source, reservoir, and tap water is 4.63 mg/l, 7 mg/l, and 0.31 mg/l, respectively (Figure 13). The reservoir sample has the highest mean value. However, according to WHO standards, there is no sulfate problem in the study area.

#### 3.1.12. Phosphate (PO_4_)

**Figure 14:**
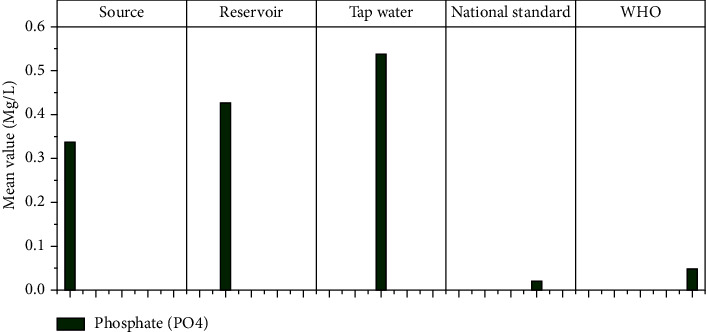
Phosphate mean values at different sampling sites.

The three most common forms of phosphorus in water are orthophosphate, condensed phosphate, and organically bound phosphate. Phosphorus is released in the form of phosphate by the microbial decomposition of organic materials. The significance of phosphorus stems from its ability to promote eutrophication in the presence of other nutrients, particularly nitrogen. The phosphorus quality criterion in water serves only to prevent undesirable algal growth [44]. The mean phosphate concentrations in this study for source, reservoir, and water tap samples were 0.38 mg/l, 0.43 mg/l, and 0.54 mg/l, respectively. Phosphate concentrations in tap water were found to be higher (0.54 mg/l). The observed value was higher than the permissible level for drinking water recommended by WHO and ES. The phosphate concentration in household tap water was higher than that in source and reservoir samples, indicating that there is phosphate ion pollution in the supply network, as shown in Figure 14. The mean phosphate value in Hawassa's water supply, on the other hand, is not significantly different from previous findings [38] in Nekemte, Oromia, and [5] in Damot Sore Woreda drinking water supply).

#### 3.1.13. Residual Chlorine

The World Health Organization recommends a minimum free chlorine residual of 0.2 mg/L and a maximum residual chlorine of 0.5 mg/L in any water supply distribution network (http://www.Safewater.Org). Several studies have discovered that when residual chlorine levels fall below recommended levels, a variety of water quality issues can occur. Bacteria and viruses known as bacteriophages can multiply in water that has not been thoroughly disinfected. It may also be capable of causing waterborne infections, depending on the species.

**Figure 15:**
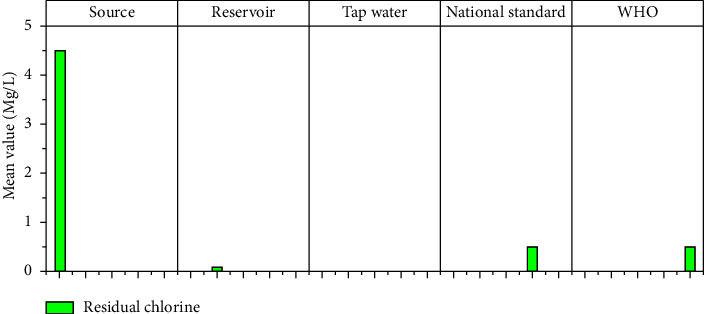
Residual chlorine mean values at different sampling sites.

The Ethiopian drinking water standard also recommends a residual chlorine level of 0.5 mg/l in drinking water. However, the mean free residual chlorine (FRC) concentration of water samples from the reservoir and the tap in this study was 0.08 mg/l and 0 mg/l, respectively (Figure 15). These values were lower than the WHO and ES maximum concentrations. This indicates that the water can be recontaminated and that there is no reserved chlorine to disinfect it, which could lead to a water-related disease in the consumer. The discovered result is also lower than the findings reported in previous studies, for example, at the Nekemte main distribution tank (0.23 mg/l and 0.28 mg/l, respectively) [38].

### 3.2. Bacteriological Analysis

**Figure 16:**
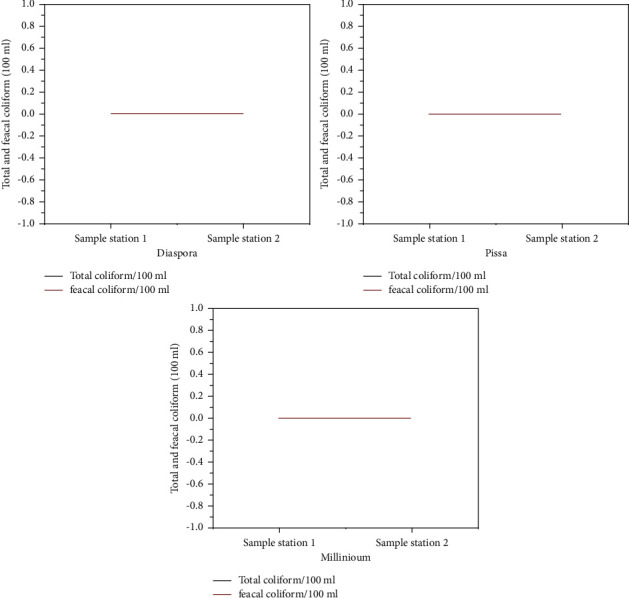
Bacteriological analysis result.

The total coliform group has been chosen as the primary indicator bacteria for the presence of pathogens in drinking water [26]. It is a primary indicator of water's suitability for consumption. If a large number of coliforms are discovered in water, it is highly likely that other pathogenic bacteria or organisms exist. Total coliform must be absent in public drinking water supplies, according to the WHO and Ethiopian drinking water feces. In this study, no coliform bacteria were found at any of the sampling sites.  Figure 16 depicts the mean total coliform bacteria levels in drinking water collected from the study area.

### 3.3. Evaluation of Water Quality Index in the Study Area

**Figure 17:**
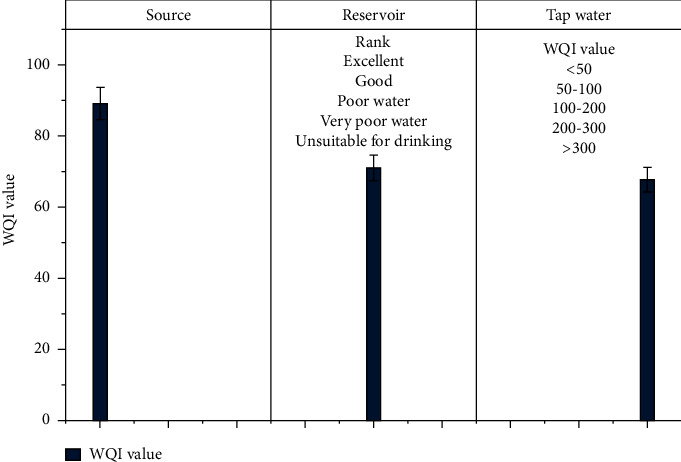
Water quality indexes result of Hawassa drinking water.

WQI is a well-known and effective tool widely used in water quality assessment [32]. Water quality data are extremely important for policy adjustment, and the water quality index (WQI) is the most convenient way to transmit the quality of drinking water resources. Several water quality indices have been developed over the years by national or international organizations and are used to assess water quality in a variety of scenarios.  Figure 17 depicts the WQI and overall WQI of all samples obtained, as determined by equations (1)–(4). According to the findings of this study, the WQI of Hawassa's drinking water supply is within acceptable limits (100). The WQI was divided into five categories, ranging from “excellent water quality” to “unfit for use water.”

The indices were developed primarily to reflect changes in the physicochemical quality of surface water. They can, however, be used as components of environmental change. There are temporal variations within an aquatic system. The system impact of this change can be measured by linking water quality to potential water use [45, 46]. In this study area, average WQI scores (ranging from 67.5 to 89) indicated that drinking water quality is good.

## 4. Conclusions

The study's goal was to assess the drinking quality of Hawassa, Ethiopia, by looking at physical, chemical, and bacteriological drinking water parameters. The drinking water quality parameters from the Hawassa City water supply's source, main reservoirs, and tap water were examined using on-site measurement and experimental analysis. The findings revealed that the majority of the water quality parameters were within the WHO and Ethiopian drinking water quality standards. Total dissolved solids (TDS), electric conductivity (EC), pH, total hardness (TH), phosphate (PO_4_), nitrate (NO_3_), sulfate (SO_4_), calcium (Ca), and magnesium (Mg) are among them. However, some physiochemical parameters (temperature, turbidity, fluoride at one well source, and residual chlorine) do not meet standards. The temperature of all water samples from the source, reservoir, and tap water exceeded 15°C. The source sample has the highest mean turbidity (24.5 NTU). However, the turbidity levels in reservoir and tap water samples are within acceptable limits (1.55 NTU and 2.48 NTU, respectively). The presence of 0.08 mg/l and 0 mg/l of free residual chlorine in tap water samples indicates that an insufficient amount of chlorine is added at the treatment plant, which could lead to recontamination of drinking water and health issues for the user. The results, on the other hand, showed that the sample analyzed was not contaminated with both total and fecal coliform, indicating that the water supply is well protected from human excreta and animal waste. In this study area, the overall average values of WQIs for source, reservoir, and tap water were 89, 71, and 67.5, respectively. As a result of the study's findings, the drinking water quality in Hawassa City can be classified as good or fair based on the water quality index classifications. Quality analysis and operational changes will be critical in improving Hawassa City's water supply system. To further guarantee that the water is fit for human use, frequent drinking water quality tests should be conducted at the source, primary distribution tanks, distribution systems, and pipelines. The investigation was limited to evaluate bacteriological and physiochemical parameters of the water delivery system from the source to household tap connections during the dry season. A comparable investigation ought to be carried out during the rainy season of the year. In addition, additional water quality factors such as heavy metals and their sources should be taken into account in future research.

## Figures and Tables

**Table 1:** Mean values and standard deviations of physicochemical parameters at the source, reservoir, and tap water samples.

Parameters	Units	11 samples from source		4 samples from reservoir		6 samples from tap water		Standard	
		Mean	Std.	Mean	Std.	Mean	Std.	ES	WHO
TDS	Mg/l	190.90	58.82	67.30	32.77	170.17	42.95	1000	1000
Temp.	°C	21.91	0.98	22.50	1.29	21.83	0.75	—	<15
EC	*μ*S/cm	339	99.68	72.75	38.39	338.67	85.17	1500	1000
Turbidity	NTU	24.45	16.21	1.55	0.45	2.48	0.38	7	5
pH	—	7.13	0.37	7.54	0.07	7.69	0.24	6.5–8.5	6.5–8.5
Ca	Mg/l	71.04	52.08	32.50	3.42	21.73	9.66	—	75
Mg	Mg/l	9.90	3.40	12.00	3.83	10.33	1.86	50	50
F	Mg/l	1.10	1.06	1.30	0.31	1.04	0.37	3	1.5
NO_3_	Mg/l	3.776	2.43	2.73	0.38	2.23	0.58	50	50
SO_4_	Mg/l	4.633	4.95	7.00	6.24	0.35	0.81	—	250
PO_4_	Mg/l	0.337	0.22	0.43	0.34	0.54	0.11	0.02	0.05
TH as CaCO_3_	Mg/l	89.864	25.95	30.00	24.15	52.50	8.22	300	300
Alkalinity	Mg/l	162.5	40.88	187.50	46.64	239.17	42.83	—	200
Residual chlorine	Mg/l	4.51	4.04	0.08	0.03	0.00	0.00	0.5	0.2–0.5

## Data Availability

The data that support the findings of this study are available from the corresponding author upon reasonable request.
